# New York Odontological Society

**Published:** 1887-01

**Authors:** 


### Reports of Society Meetings.

### NEW YORK ODONTOLOGICAL SOCIETY.

REPORTED FOR THE INDEPENDENT PRACTITIONER.

The first meeting of this society for the season was held in the parlors of the Academy of Medicine, on Tuesday evening, Oct 12th, Vice-President Dr. J. Morgan Howe in the chair.

The meeting was largely attended, and its interest greatly augmented by an address from Dr. William J. Younger, of San Francisco, on the subject of tooth implantation. The doctor described at length his methods of operating in implanting, transplanting and replanting teeth, also citing a number of interesting cases in which such operations had been successfully performed.

The members of the society were particularly favored in having present one of Dr. Younger's patients, who happened to be in New York at that time, and who kindly consented to attend the meeting, thus giving those present an opportunity to view and examine what seemed to be a successfully performed operation. The patient, a lady in middle life, had a left superior central incisor which, from pyorrhœa, had become so loose as to drop down upon the lower lip. This, together with the superior left lateral incisor, which also had become diseased, was removed by Dr. Younger, and the alveoli deepened, obliterating all traces of the disease. The teeth were opened from the foramen and the canal contents entirely removed, and after being disinfected in a bath of bi-chloride of mercury, were filled with gutta-percha to within a short distance from the apex, the remaining space being filled with gold. The ends were then slightly ground, smoothly polished, placed in position in their newly prepared sockets and ligated.

Some years previous to this operation this patient had lost a right superior second bicuspid, and in the space Dr. Younger separated the gum and cut into the bone, forming a new alveolus for the reception of a tooth. One of the proper size and shape having been selected and properly prepared (similar to the other cases mentioned), it was forced into the new opening, where it now remains fixed and firm.

Vice-President Howe appointed a committee of three, naming Drs. Atkinson, Jarvie and Lord, to carefully examine the teeth in the lady's mouth and report to the society, the members taking a recess during the interim. After a careful examination by these gentlemen, Dr. Atkinson introduced the small blade of a pen-knife between the gum and neck of the bicuspid, producing slight pain and starting a little blood. He expressed himself highly pleased with the appearance of the teeth, and believed them firmly attached. Dr. Jarvie stated that, so far as he was able to judge by examination with the mouth-mirror and testing their stability with his fingers, they were as firm in their attachment as any teeth in the lady's mouth.

Dr. Younger was asked how long a time the implanted bicuspid had been out of the mouth from which it was taken, and replied that he could not say with certainty, but it might have been weeks or months. This, however, he considered immaterial. His theory is that, however dry may be the investing membrane of the root, it still retains a germ of life, and, much like seeds, requires only proper nourishment to become re-vivified.

Dr. Carl Heitzmann and others expressed adverse opinions regarding the re-vitalizing of the pericementum, believing that, with the extraction and drying of a tooth, the membrane forever loses its vitality. It was also stated that, even if the dried membrane retained dormant life, the immersion of the tooth in a solution of bichloride of mercury would surely destroy it. The idea was advanced by a member that, even though the pericementum was devoid of vitality when placed in the newly-prepared socket, living matter might penetrate its meshes and thus form an attachment between tooth and alveolus, on the principle of sponge-grafting.

Dr. Weld stated that a few years ago he was quite as enthusiastic as Dr. Younger now is on the subject of transplantation and replantation of teeth, and had performed such operations in many instances, often attaching porcelain crowns to natural roots. He had been careful to preserve the membranes surrounding the roots, but of all the cases he had treated, he believed that few, if any, could be considered permanent, or could be termed an entire success. He predicted for Dr. Younger the disappointment and ultimate failures he himself had experienced.

In reply to a question asked, Dr. Younger said that he had patients in the west for whom he had performed these operations years ago, and teeth thus inserted remained firm to the present time, and notwithstanding Dr. Weld's prediction of failures, he was satisfied with the results of his operations.

Dr. A. H. Brockway was pleased to have seen the result of the operation as presented in the mouth of Dr. Younger's patient, who was present at the meeting. He stated that good dentists put good fillings into teeth, yet some of these might keep the cavities from decay for only three or four years, and then come out. He would not consider such cases exactly in the light of failures. It is so with the operations described by Dr. Younger. If they are the means of saving our patients from the necessity of wearing plates of artificial teeth for a number of years, they were of benefit, and should not be classed as failures.

Two patients were also at the meeting for whom Dr. Younger had implanted teeth a few days before, and the operations seemed to indicate successful results.

A vote of thanks was tendered to Dr. Younger for his comprehensive paper, and the explanations following; also to his lady patient for so kindly submitting her teeth to the inspection of members of the society.

### MEETING OF NOVEMBER 9TH.

The November meeting of this society was held at the rooms of the N. Y. Academy of Medicine on the 9th, Vice-President J. M. Howe occupying the chair.

Dr. W. G. A. Bonwill, of Philadelphia, the essayist of the evening, read an interesting paper, reviewing the various methods in vogue for condensing gold foil. His text seemed to be "Rotation *versus* Filling by Hand."

The method of consolidating gold foil by revolving instruments, as introduced by Dr. Herbst, was so antagonistic to his idea and method of filling that he was induced to give much time and attention to the inspection of this method of practice, and he was therefore a close observer of Dr. Herbst's manipulations in various places where he was giving clinics. Asking himself this question—"What do I think of Dr. Herbst?" the essayist replied by saying that he had been anxious to see him and to inspect his methods, which were so opposed to those cherished by himself. All were able to see what had been produced by the various methods previously adopted for filling cavities, "and how many," asked the doctor, "think former methods have all proved failures ? Have we just awakened to find the old ways faulty ?" He would give credit to the brother from the Fatherland who, when laboring under great difficulties and working against adverse circumstances, had, by persistent effort, accomplished so much, and who had so generously imparted to others the knowledge thus acquired.

Dr. Bonwill also referred to the modest bearing of Dr. Herbst, who, during his visit among us, stated that if he could have foreseen what he had since witnessed in the operations of American dentists, he would never have ventured here to demonstrate his method. "Are we now," asked Dr. Bonwill, "prepared to recognize this new method as the best ?" In accomplishing good results, does the success depend on materials or men ? Dr. Herbst knew the peculiarities or ways of gold, and how to render it pliable; also how best to adapt means to ends.

Dr. Bonwill claimed that his method of condensing gold with the mechanical mallet made perfect adaptation with less labor, and insured better results. It was pleasanter for patient as well as operator, and in every respect more satisfactory. He deemed practicability and adaptability the essential requisites to be considered. He discovered that Dr. Herbst did not consolidate the foil solely by rotation, but much of his work was done by hand-pressure instruments, and he tested every part of each filling with sharp points to make sure that the gold was properly condensed. Dr. Bonwill would not like to spend so much time in packing by hand-pressure and then condensing by rotation, neither did he like to retain the thin cavity margins which are so likely to break away. He would prefer a surplus of metal to overlap the edges, which could be nicely and securely trimmed away in finishing.

He did not consider clinical operations a fair test of an operator's ability, on account of the disadvantages under which he labors. He thought a good operator should pursue a system and see the end from the beginning. To forestall the attack of caries in future, he would not resort to the Herbst method of preparing cavities. He considers the saving of time an object in dental operations, and, furthermore, the average operator would not be able to succeed well in adopting the rotation method. He believes that dental operations are constantly improving in character. He would not give up the mechanical plugger. If it did not do perfect work it was the fault of its manipulator, and not the instrument. He would not lay it aside for any new appliance, feeling a sense of greater security in his old boat which had safely carried him over stormy seas. He thought few fully understood the working of the various appliances. He considers it impossible to slide the gold against the walls of the cavity by single blows, and believed that the pushing blows of the automatic spring mallet gave a wrong impact. He contends that we need an instrument that will slide smoothly and can be easily guided; one that will carry the gold back and forth across the cavity and well against the walls with lateral movements, leaving it concave in the center of the cavity, and working each way until the walls are secure, then completing the operation by rounding out the center. He suggests soft foil for lining cavity walls, and small points for condensing, the whole being finished with cohesive gold.

The instruments devised by Dr. Bonwill have rounded working tips and are finely serrated, so that in pushing or pressing they carry the foil before them, instead of cutting or tearing it. He is in the habit of cutting the foil into strips, and with his engine mallet lapping their flat surfaces together in the cavity. He believes in light blows, given with rapidity. He is careful, not only in filling cavities, but equally so in their preparation, looking particularly to smooth walls. If necessary to anneal his gold, he does not carry it directly in the flame, but heats it on a thin plate of platinum.

He has struggled for years to acquaint the profession with his mallet and his method of filling teeth, and he was glad to notice that his efforts were showing signs of appreciation.

Upon the completion of his paper, Dr. Bonwill passed around the room for inspection some of his fillings which had been removed from cavities in which they had been impacted, and again beaten into foil. The specimens were conveyed between sheets of glass.

A vote of thanks from the society was given to Dr. Bonwill for his paper, and it was moved that, owing to its length, it be discussed at the next meeting of the society, and that the author be invited to attend.

After the motion was carried, Vice-President Howe asked Dr.

Bonwill if he would exhibit to the society his new tooth brush, and say a few words regarding its merits.

Dr. Bonwill passed a number of the brushes to the members, but owing to the lateness of the hour reserved his remarks for a future occasion.

Dr. Beverly Cole, of San Francisco, being present, was introduced to the society, and stated that although not a dentist he had been much interested in listening to the paper read and the remarks following. He had been quite a traveler in this and other countries, and had found that on the other side of the ocean American dentistry was admitted to be by far the most advanced in the world. In medicine, however, the Germans were considered to be ahead, but after visiting many German hospitals he felt convinced that this advance was in theory only, for students were led from bedside to bedside, and with pencil and slate noted the muscular movements of each patient and their general condition, then were passed to the next in turn without attention to remedial measures. He considered the young American physician, as is everything else American, to be practical, and while carefully noting the conditions of their patients, they at once administer to their relief or operate for their recovery. Dr. Cole said that if his life was endangered by illness, he would trust it to the care and keeping of the young American physician in preference to all others.

